# Editorial: Anticancer Potential of *Artemisia annua*

**DOI:** 10.3389/fonc.2022.853406

**Published:** 2022-02-24

**Authors:** Jill M. Kolesar, Peter H. Seeberger

**Affiliations:** ^1^ Department of Pharmacy Practice and Science, College of Pharmacy, University of Kentucky, Lexington, KY, United States; ^2^ Department of Biomolecular Systems, Max-Planck Institute of Colloids and Interfaces, Potsdam, Germany

**Keywords:** Cancer, Artemisia annua, Artesunate, Artemisinin, dihydroartemisin

Extracts of *Artemisia annua* and related plants have been used for centuries to treat and prevent malaria as well as other infectious diseases caused by parasites, viruses, and bacteria (1). The identification of the active pharmaceutical ingredient artemisinin eventually resulted in the preparation of the semi-synthetic derivative artesunate that has a longer half-life and better bioavailability when compared to artemisinin (2). Following the discovery that artemisinin and its derivatives are active against a host of human cancer cell lines by Prof. Efferth in 2001 (3), preclinical and clinical evidence has been obtained to make a strong case for significant anticancer activity for artemisinin derivatives such as artesunate (4). However, the pharmacological challenges such as short half-life and poor bioavailability may limit anticancer activity (5). In this Research Topic, Kagan et al. report the development of 2-carbon-linked dimeric artemisinins (2C-ART) that demonstrate low-nanomolar IC50s against multiple AML cell lines. The lead compound, ART631, elevated ROS and induced apoptosis and was active in combination with sorafenib and venetoclax in both cell models and xenografts. While ART631 is limited by less than optimal *in vitro* stability, further optimization to improve efficacy and stability are warranted.

A wide variety of anticancer mechanisms have been proposed for artemisinins, including inducing apoptosis, cell cycle arrest, ferroptosis and inhibiting angiogenesis and invasion (4). While multiple signaling pathways are implicated, few direct targets for artesunate are reported. Li et al. for the first time demonstrate that human telomerase reverse transcriptase (hTERT) is overexpressed in esophageal cancer compared to adjacent normal tissue and correlated with poor prognosis. They also demonstrate that dihydroartemisinin (DHA), which is a semi-synthetic derivative of artemisinin, reduces proliferation of esophageal cancer cells and down regulates hTERT expression at the transcriptional level *via* interaction and down regulation of the transcription factor SP1. DHA also reduced tumor volumes in xenograft models. This is a novel mechanism of action for DHA, which may help guide further study into synergistic combinations for the treatment of esophageal cancer.

Yi et al. have also identified a novel mechanism of DHA activity in colon cancer. RNA seq analysis between DHA treated and untreated cancer cells suggested overexpression of CDK1, CCNB1 and PLK1, which was followed with molecular docking studies that suggest that DHA could dock into the CDK1/CCNB1 complex resulting in cell cycle arrest in the G2/M phase and suppressed CDK1/CCNB1/PLK1 signaling activation. While artemisinin derivatives are well known to affect the cell cycle, this report is the first to identify the CDK1/CCNB1 complex as a potential target.

This Research Topic provides insights into novel artemisinin derivatives as well as proposes several new mechanisms of action which we anticipate will help investigators in the field advance their studies. However, several key gaps in knowledge still exist. First, the specific anticancer mechanism of artemisinins remains elusive, with dozens of potential targets and mechanisms demonstrated across a variety of cell lines. A unifying mechanism of action (or disproval) still remains to be identified. Next, artemisinin derivatives remain pharmacologically challenging compounds with short *in vivo* half-lives and relatively low potency, representing further opportunities for compound optimization. Finally, identification of mechanisms of artesunate resistance remain in their infancy and are critical to the ultimate development of these artemisinin derivatives as anticancer agents (6).

## Author Contributions

JK: Initial draft, review, editing, and approval of final version. PS: Review, editing, and approval of final version. All authors contributed to the article and approved the submitted version.

## Conflict of Interest

JK: Patent pending related to artemisinins. PS: Patent pending related to artemisinins and ownership interest in ArtemiLife.

## Publisher’s Note

All claims expressed in this article are solely those of the authors and do not necessarily represent those of their affiliated organizations, or those of the publisher, the editors and the reviewers. Any product that may be evaluated in this article, or claim that may be made by its manufacturer, is not guaranteed or endorsed by the publisher.
